# Potential of Proton Therapy to Reduce Acute Hematologic Toxicity in Concurrent Chemoradiation Therapy for Esophageal Cancer

**DOI:** 10.1016/j.ijrobp.2017.07.025

**Published:** 2017-11-01

**Authors:** Samantha Warren, Christopher N. Hurt, Thomas Crosby, Mike Partridge, Maria A. Hawkins

**Affiliations:** ∗ Cancer Research UK/Medical Research Council Oxford Institute for Radiation Oncology, Gray Laboratories, University of Oxford, Oxford, United Kingdom; † Wales Cancer Trials Unit, School of Medicine, Cardiff, United Kingdom; ‡ Velindre Cancer Centre, Velindre Hospital, Cardiff, United Kingdom

## Abstract

**Purpose:**

Radiation therapy dose escalation using a simultaneous integrated boost (SIB) is predicted to improve local tumor control in esophageal cancer; however, any increase in acute hematologic toxicity (HT) could limit the predicted improvement in patient outcomes. Proton therapy has been shown to significantly reduce HT in lung cancer patients receiving concurrent chemotherapy. Therefore, we investigated the potential of bone marrow sparing with protons for esophageal tumors.

**Methods and Materials:**

Twenty-one patients with mid-esophageal cancer who had undergone conformal radiation therapy (3D50) were selected. Two surrogates for bone marrow were created by outlining the thoracic bones (bone) and only the body of the thoracic vertebrae (TV) in Eclipse. The percentage of overlap of the TV with the planning treatment volume was recorded for each patient. Additional plans were created retrospectively, including a volumetric modulated arc therapy (VMAT) plan with the same dose as for 3D50; a VMAT SIB plan with a dose prescription of 62.5 Gy to the high-risk subregion within the planning treatment volume; a reoptimized TV-sparing VMAT plan; and a proton therapy plan with the same SIB dose prescription. The bone and TV dose metrics were recorded and compared across all plans and variations with respect to PTV and percentage of overlap for each patient.

**Results:**

The 3D50 plans showed the highest bone mean dose and TV percentage of volume receiving ≥30 Gy (V_30Gy_) for each patient. The VMAT plans irradiated a larger bone V_10Gy_ than did the 3D50 plans. The reoptimized VMAT62.5 VT plans showed improved sparing of the TV volume, but only the proton plans showed significant sparing for bone V_10Gy_ and bone mean dose, especially for patients with a larger PTV.

**Conclusions:**

The results of the present study have shown that proton therapy can reduced bone marrow toxicity.

**Summary**

Radiation therapy dose escalation is predicted to improve local tumor control in esophageal cancer; however, any increase in acute hematologic toxicity could limit the predicted improvement in patient outcomes. We investigated the bone marrow dose of volumetric modulated arc therapy (VMAT), proton therapy, and bone marrow-sparing VMAT plans for esophageal tumors. Improved bone marrow sparing was possible with VMAT, but only proton therapy resulted in significant sparing for bone V_10Gy_ and bone mean dose, especially for patients with larger planning target volumes.

## Introduction

Esophageal cancer is the sixth most common cause of death worldwide, representing 5% of total cancer deaths in 2012 (1). Although surgery offers the best outcomes for patients with esophageal cancer, only 10% to 20% of patients with nonmetastatic disease will be eligible for surgical treatment. Therefore, definitive concurrent chemoradiation therapy has been recognized as a valuable treatment option for many patients. Also, the recent SCOPE1 trial (ISRCTN number 47718479) reported good outcomes in the standard definitive concurrent chemoradiation therapy arm (cisplatin/5-fluorouracil [5-FU] with 50 Gy in 25 fractions) with a 2-year overall survival rate of 56%, with hematologic toxicity (HT) grade ≥3 the most commonly reported acute side effect (28% of patients) (2). Only 53% of the patients completed chemotherapy at the full dose (2). Radiation therapy dose escalation using a simultaneous integrated boost (SIB) is predicted to further improve local tumor control in these patients (3). Treatment planning studies for patients with mid-esophageal cancer suggested that a boost dose of 62.5 Gy in 25 fractions could be achieved using either photons (4) or protons (5) without exceeding the dose constraints for the heart, spinal cord, and lungs. A pilot study treating 25 patients with photons has described safe escalation of 62.5 Gy in 25 fractions as a boost to a positron emission tomography (PET)-guided gross tumor volume (GTV) (6). In contrast, any increase in the radiation dose could cause an increase in HT owing to greater bone marrow irradiation. This could reduce the treatment intensity by interrupting delivery or reducing the dose of concurrent chemotherapy, limiting any predicted improvement in patient outcomes.

Studies of patients who underwent fractionated radiation therapy have indicated that at less than ∼50 Gy, bone marrow has a large capacity for repair and regeneration (although this could require many months or even years) (7). The findings from longitudinal ^18^F-fluorothymidine (FLT)-PET imaging of pelvic cancer patients have suggested that acute HT can occur at low doses (4 Gy), with bone exposed to >35 Gy exhibiting chronic toxicity, with reduced bone marrow recovery 1 year after treatment (8).

Most of the data concerning the risk of HT and bone marrow irradiation have been collected from studies of anal cancer (9) or cervical cancer 10, 11, in which the large planning treatment volumes (PTVs) often abut or overlap the pelvic bones (pelvis, sacrum, lumbar spine). The pelvic bones, combined, contain ∼50% of the body's active bone marrow. Dose–volume metrics such as the mean pelvic bone dose and pelvic bone V_10Gy_ and V_20Gy_ (percentage of volume receiving ≥ *x*Gy) have been linked to the risk of grade ≥2 leukopenia and neutropenia (10), when the external contour of the pelvic bony structures was used as a surrogate for bone marrow irradiation. Intensity modulated radiation therapy techniques (intensity modulated radiation therapy [IMRT] and volumetric modulated arc therapy [VMAT]) can be used in the treatment of these tumor sites for improved pelvic bone marrow sparing. Reduced HT in cervical cancer (12) and reductions in HT for anal cancer (13) treated with IMRT and scanned proton therapy (14) have been reported. These delivery techniques can also be combined with functional imaging to identify active bone marrow regions within the pelvis to guide dose optimization 11, 15, 16. The dose to subsites of the pelvic bone (eg, lumbosacral spine, which contains 25% of the bone marrow) has also been identified as an important predictor of HT (17), and preferential dose sparing to a subregion of pelvic bone might be more easily achieved.

HT is also observed in patients undergoing chemoradiation therapy for thoracic malignancies 2, 18, 19. Approximately 35% of the active bone marrow is found in the thoracic vertebrae (VT) (20). A reduced proliferation of irradiated bone marrow in the thoracic spine after only 2 Gy has also been detected using FLT-PET imaging for lung cancer patients undergoing chemoradiation therapy (21).

A recent study of 52 lung cancer patients treated with 3-dimensional (3D) conformal radiation therapy and IMRT found the TV dose parameters (mean dose, V_20Gy_, and V_30Gy_) were associated with the risk of grade ≥3 leukopenia (22). A study of 41 esophageal cancer patients investigated similar dose–volume parameters (TV mean dose, V_20Gy_, and V_10Gy_) to propose cutoff values to avoid the development of grade ≥3 leukopenia (23). The size and position of the target volumes also means that the potential for dose sparing of the bone marrow in the thorax might be greater than that for the pelvis, especially if IMRT, VMAT, or proton therapy is used. Although these techniques might improve the conformality of the high-dose region, the distribution of low doses (5-15 Gy) is very different with IMRT or VMAT compared with 3D conformal radiation therapy. Proton therapy plans have demonstrated a reduction in bone V_10Gy_ by 30% and 27% compared with 3D50 and IMRT plans, respectively (24). Also, reduced HT for lung cancer patients treated with passive scattering proton therapy has been reported (25).

Given the proximity and anatomic location of the esophagus in relationship to the vertebral bodies and the technological capabilities of modern linear accelerators, the potential for bone marrow sparing for individual patients also merits further investigation.

Therefore, we compared the dose–volume metrics for thoracic bone structures to identify differences in the dose distribution for a representative group of patients with mid-esophageal cancer from conformal, VMAT, and proton therapy techniques. The potential for reoptimizing VMAT plans to improve bone marrow sparing or selecting patients for proton therapy is also illustrated by comparing the dosimetric parameters for individual patients.

## Methods and Materials

The data from 21 patients who had undergone definitive chemoradiation therapy for mid-esophageal cancer from the SCOPE1 clinical trial previously used to model dose escalation and proton treatment delivery were further investigated 4, 5. These patients, with a mean PTV of 327 cm^3^ (range 140-591) had been randomly selected previously 4, 5 as a representative subset of the entire SCOPE1 trial database, for which the mean PTV was 334 cm^3^.

The trial-derived GTVs were used for each patient, and each GTV was extended along the esophagus manually by ±2 cm cranially and caudally. The clinical target volume (CTV_50Gy_) was created using an additional radial margin of 1 cm, and no elective nodal irradiation was given. Another 1-cm margin was added to generate the PTV_50Gy_, which received a dose prescription of 50 Gy in 25 fractions for the standard dose plans. For the plans with a SIB dose prescription of 62.5 Gy in 25 fractions, the CTV_62.5Gy_ was considered identical to the GTV, and the PTV_62.5Gy_ was then generated using an isotropic margin of 0.5 cm to allow for dose fall off from 62.5 to 50 Gy.

A surrogate for bone marrow (bone) was created by outlining the TV (T1-T12 inclusive), sternum, scapulae, ribs, and clavicles using the automatic thresholding tool in Eclipse (Varian Medical Systems, Palo Alto, CA). A separate volume for the body of the thoracic vertebrae T1 to T12 was created (TV). The dose calculated from the 3D conformal plan (3D50), typically of 4 fields (anteroposterior and lateral–oblique) was available and was used to record the dose to the bone and TV.

Additional treatment plans were created retrospectively in Eclipse using the VMAT technique and, for dose-escalated plans, using VMAT and proton therapy. All arc therapy plans consisted of 2 complete 360° arcs (clockwise and counterclockwise) of 6 MV. Initially, 1 plan with the same standard dose prescription used in the original treatment plan (VMAT50) and a dose-escalated VMAT plan (VMAT62.5) with a SIB dose prescription of 62.5 Gy in 25 fractions to the high-risk subregion within the PTV were created (4).

**Fig. 1:**
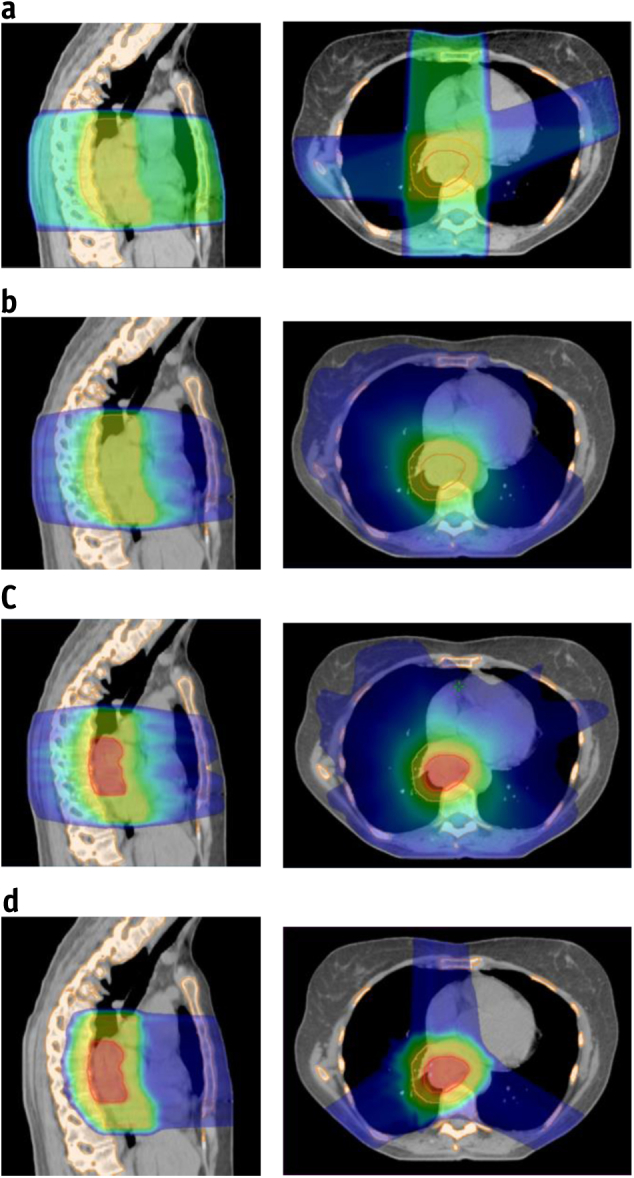
Dose distributions for a typical mid-esophageal cancer patient illustrating (a) 3-dimensional conformal plan (3D50), (b) standard-dose volumetric modulated arc therapy (VMAT) plan (VMAT50), (c) dose-escalated VMAT plan (VMAT62.5), and (d) dose-escalated spot-scanning proton plan (SFO62.5). Bone outlined in orange, planning treatment volume to 50 Gy (PTV_50Gy_) in yellow, and PTV_62.5Gy_ in red. The dose color wash is from 10 Gy (blue) to 40 Gy (green) to 65 Gy (red). (A color version of this figure is available at www.redjournal.org.)

A 3-field spot-scanning proton therapy plan with 70 to 250 MeV using single-field optimization (SFO62.5) was created with the same SIB dose prescription using an anterior and posterior–oblique beam arrangement to improve heart and lung sparing (26). Additional field-specific proximal and distal margins of 0.3 to 0.5 cm were applied to the PTV_50Gy_ to account for a 3.5% range error (5). The same dose–volume constraints for plan optimization, target coverage, and dose to the organs at risk (heart, lung, spinal cord) were initially applied to the VMAT and proton plans, and no explicit dose constraints for bone marrow sparing were used initially. The dose distributions obtained with these 4 plans are shown in Figure 1.

Subsequently, a dose-escalated and bone marrow-sparing VMAT plan was created for each patient to reduce the dose to the bone marrow (VMAT62.5bm). Using all the thoracic bone structures (T1-T12, sternum, scapulae, ribs, and clavicles) as an optimization volume was deemed impractical. Thus, the dose reduction was applied only to the TV volume nonoverlapping with the PTV using a mean dose constraint and additional dose–volume constraints in the 10- to 30-Gy region for the TV substructure. The proton plans were not reoptimized for bone marrow sparing, given the sharp dose falloff around the target adjacent to the TV volume and the already low dose to the other thoracic bone structures.

For all the plans, the bone mean dose, bone V_20Gy_, bone V_10Gy_, TV mean dose, TV V_20Gy_, TV V_10Gy_, lung mean dose, lung V_20Gy_, heart mean dose, and heart V_30Gy_ dose metrics were recorded. Tests for statistical significance were performed in SPSS, version 20 (IBM Corp., Armonk, NY) using the Wilcoxon signed rank test for pairwise comparisons of the dose–volume metrics for the different irradiation techniques. To better understand the parameters (eg, PTV, number of vertebrae irradiated [NV], percentage of TV overlap) that might affect the potential for bone marrow sparing when reoptimizing the VMAT62.5 plans, the differences in the dose–volume metrics achieved for SFO62.5 versus VMAT62.5 and SFO62.5 versus VMAT62.5bm were examined for the individual patients to assess the benefits of proton therapy for improving bone marrow sparing. These were tested using the Pearson correlation.

## Results

**Fig. 2:**
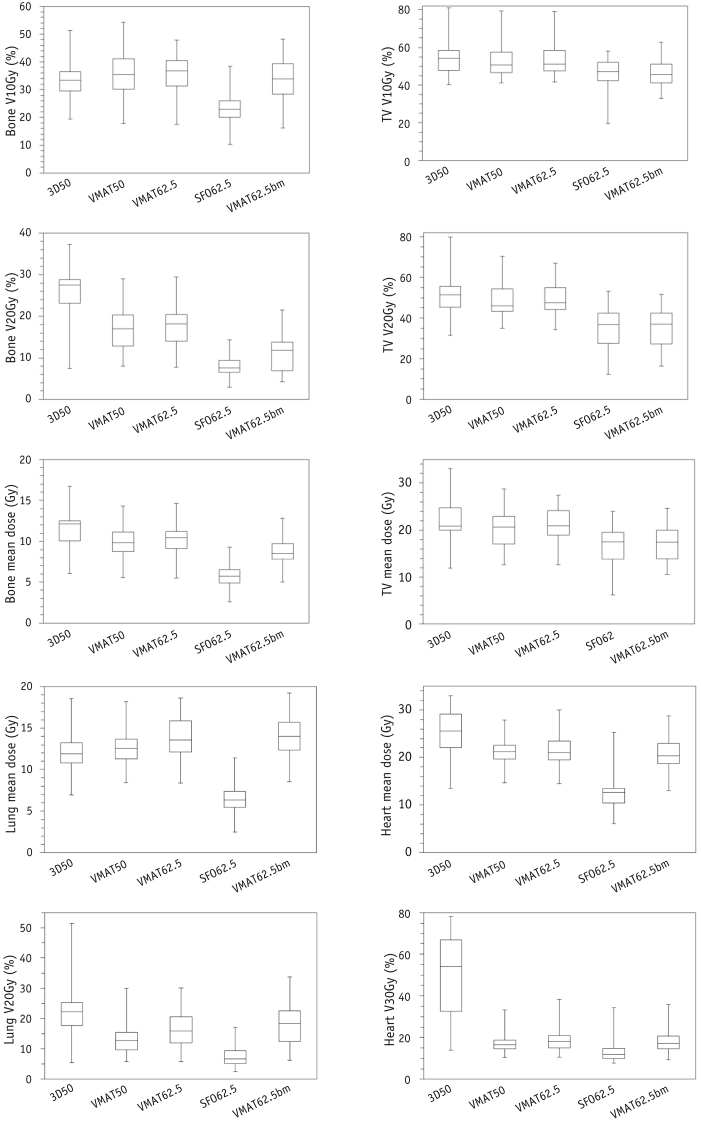
Box plots comparing the dose–volume parameters for bone, thoracic vertebrae (TV), heart, and lung obtained for 3-dimensional conformal (3D50), volumetric modulated arc therapy to 50 Gy (VMAT50), dose-escalated VMAT62.5, dose-escalated single-field optimization (SFO62.5), and VMAT62.5bm (optimized for bone marrow sparing) treatment plans for the 21 patients.

The dose distribution data were compared quantitatively for all patients for each technique (Fig. 2; Table E1; available online at www.redjournal.org). The 3D50 plans generated the highest bone mean dose (median 12.1 Gy, interquartile range [IQR] 10.1%-12.5%) and bone V_20Gy_ (median 27.5%, IQR 23.2%-28.8%). No clinically significant difference (ie, <2 Gy in absolute dose or <5% in V_*x*Gy_) was found between the original VMAT_50_ and VMAT_62.5_ plans for any of the dosimetric parameters, suggesting that dose escalation with VMAT could be achieved without a clinically significant increase in bone marrow irradiation for all patients. Both original VMAT plans generated a slightly greater median bone V_10Gy_ (VMAT50 median 35.3%, IQR 30.1%-41.0% and VMAT62.5 median 37.9%, IQR 31.6%-41.8%) than did the 3D50 plans (median bone V_10Gy_ 33.4%, IQR 29.5%-36.4%; Wilcoxon signed rank test, Z = −2.172, *P*=.030 compared with VMAT50). No clinically significant difference was found in the TV mean dose between the photon techniques, and differences in TV V_20Gy_ were modest between the 3D50 (median 51.5%, IQR 45.5%-57.0%) and VMAT plans (VMAT50 median 46.0%, IQR 43.3%-54.4% and VMAT62.5 median 47.5%, IQR 44.5%-55.3%). The median TV V_10Gy_ for this group of patients was similar to that of the 3D50 plans (median 54.3%, IQR 47.9%-58.5%), VMAT50 plans (median 50.7%, IQR 46.6%-57.5%), and VMAT62.5 plans (median 51.0%, IQR 47.7%-58.4%), although the VMAT62.5bm plans showed a small reduction in TV V_10Gy_ (median 45.5%, IQR 41.2%-51.1%). This was similar in magnitude to the TV V_10Gy_ sparing with the SFO62.5 plans (median TV V_10Gy_ 47.1%, IQR 42.5%-52.1%).

In contrast, SFO62.5 showed clinically significant sparing (>2 Gy in absolute dose or >5% in V_*x*Gy_) for many bone volume parameters compared with the photon techniques, notably for the bone mean dose (median 5.7 Gy, IQR 4.9-6.5) and bone V_10Gy_ (median 23.0%, IQR 20.0%-26.0%). In contrast to the VMAT62.5 plans, the TV mean dose was reduced by >3 Gy (median 17.6 Gy, IQR 15.0-19.8) for the proton planning technique. These differences in the SFO62.5 versus VMAT62.5 plans were also highly statistically significant (Z = −4.015; *P*<.001). However, for the overall group of patients, the SFO62.5 plans had TV V_10Gy_ values (median 47.1%, IQR 42.5%-52.1%) similar to those of the VMAT62.5bm plans, indicating that the smaller TV and PTV overlap can also be beneficial for TV sparing in the VMAT62.5bm plans. Nevertheless, the minimum TV V_10Gy_ value (19.5%) for patients with the smallest TV overlap in the PTV was much smaller for the proton plans than for the VMAT62.5bm plans (33.0%).

The dose–volume parameters for the heart and lung are also shown in Figure 2. These indicated that dose escalation can be achieved with only a small increase in the mean dose to lung and that proton plans produce significant sparing of the heart, lung, and bone marrow structures compared with VMAT and conformal techniques, as described previously (5).

**Fig. 3:**
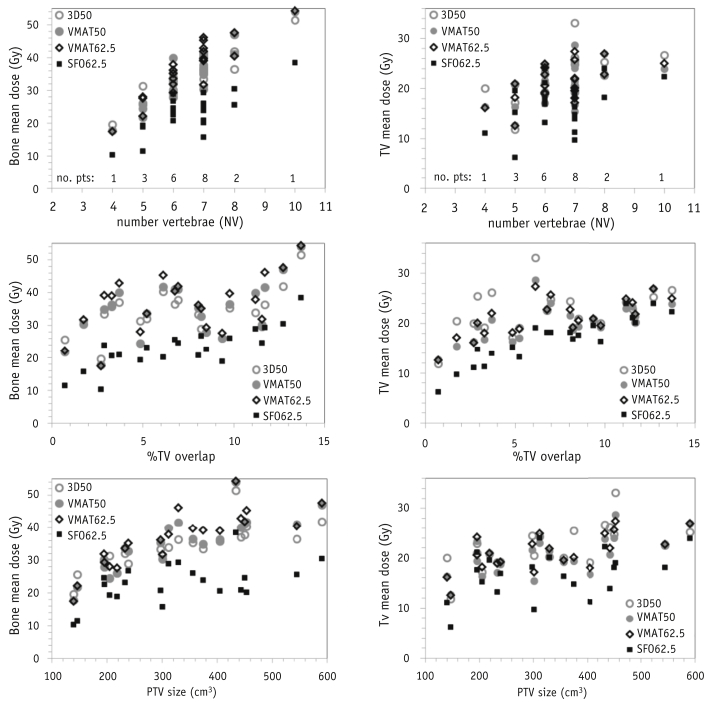
A comparison of bone and thoracic vertebrae (TV) mean dose for individual patients as a function of number of vertebrae (NV), planning treatment volume, or percentage of TV overlap. Dose metrics for 3-dimensional conformal (3D50; open gray circles), standard volumetric modulated arc therapy (VMAT50; open gray triangles), dose-escalated VMAT (VMAT62.5; open black squares); and dose-escalated single-field optimization (SFO62.5; solid black diamonds) plans shown.

Notwithstanding the overall trends observed, considerable variation was found in the bone marrow dose–volume metrics for individual patients (Fig. 3). The correlation of mean dose for bone and TV with factors such as the NV irradiated, percentage of TV overlap in the PTV (median overlap 7.0%, IQR 5.0%-10.5%), and PTV was also examined. For both bone and TV, although a general trend was seen toward a higher dose with a larger NV, this is not a sufficiently unique value to clearly differentiate patients at the greatest risk of higher bone marrow irradiation, regardless of the technique used.

Variations in the bone mean dose (and bone V_20Gy_ and V_10Gy_; data not shown) with the different treatment techniques appeared most clearly linked to the PTV. For the bone mean dose, a general increase in dose with PTV was seen for the 3D50, VMAT50, and VMAT62.5 plans. For 16 of 21 patients, the V_10Gy_ dose was greater with the VMAT50 plans than with the 3D50 plans owing to the increased low-dose bath generated by the arc therapy technique. Only the proton plans were able to reduce the dose–volume metrics for all the patients compared with all the 3D50 and VMAT plans, in particular, when the PTV was >300 cm^3^.

The trend toward a greater TV mean dose (and TV V_20Gy_ and TV V_10Gy_; data not shown) with a larger overlap was more pronounced for the proton plans. Thus, for this series of patients, proton therapy might have a limited ability to spare the TV with an overlap greater than ∼8%.

The results when applying the TV dose–volume cutoff values to predict the risk of grade ≥3 leukopenia proposed by Lee et al (23) and Deek et al (22) are listed in Table 1, including the number of patients (absolute and percentage of group) exceeding the threshold values for each treatment technique. The cutoff values proposed by Lee et al (23) and Deek et al (22) are different, but the trends observed were the same. The SFO62.5 and VMAT62.5bm plans showed the lowest risk of HT, which was greatly reduced compared with the 3D50, VMAT50, and standard VMAT62.5 plans.

**Table 1:** Patients predicted to be at risk of grade ≥3 leukopenia using threshold dose–volume metrics for thoracic vertebrae from Lee et al (23) and Deek et al (22)

Grade ≥3 leukopenia cutoff	Treatment plan				
	3D50	VMAT50	VMAT62.5	SFO62.5	VMAT62.5bm
Lee TV mean <25.9 Gy	3 (14)	2 (10)	2 (10)	0 (0)	0 (0)
Lee TV V_20Gy_ <70%	1 (5)	1 (5)	1 (5)	0 (0)	0 (0)
Lee TV V_10Gy_ <77%	1 (5)	1 (5)	0 (0)	0 (0)	0 (0)
Deek TV mean <23.9 Gy	8 (38)	3 (14)	6 (29)	2 (10)	1 (5)
Deek TV V_20Gy_ <56.0%	5 (24)	4 (19)	4 (19)	0 (0)	0 (0)
Deek TV V_30Gy_ <52.1%	6 (29)	2 (10)	2 (10)	0 (0)	0 (0)

*Abbreviations:* 3D50 = 3-dimensional conformal radiation therapy; bm = bone marrow sparing (plan); SFO = single-field optimization; TV = thoracic vertebrae; V_*x*Gy_ = percentage of volume receiving ≥ X Gy; VMAT = volumetric modulated arc therapy.

Data presented as absolute number of patients and percentage of the 21 patients included in the present study.

**Fig. 4:**
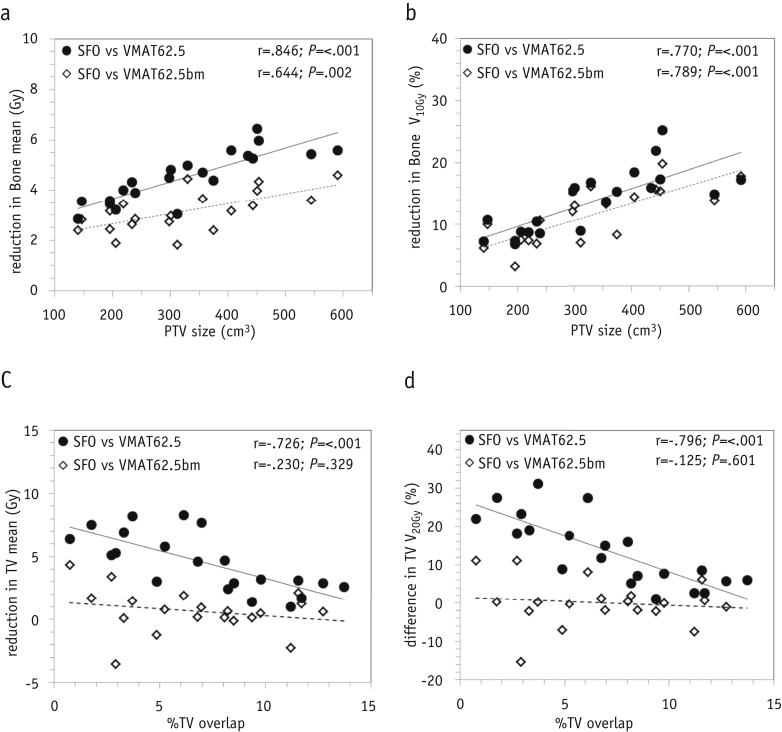
A comparison of dose–volume metrics for individual patients for bone as a function of (a,b) planning treatment volume (PTV) and (c,d) thoracic vertebrae (TV) as a function of the percentage of TV overlap. Solid circles indicate differences in the dose-escalated single-field optimization (SFO62.5) and original dose-escalated volumetric modulated arc therapy (VMAT62.5) plans; open diamonds, differences with the reoptimized bone marrow-sparing VMAT62.5bm plans.

When reoptimizing the VMAT plans to explicitly spare the bone marrow, the PTV, percentage of overlap of the TV, and proximity and overlap of the PTV with other organs at risk (eg, heart, lung, and spinal cord) were critical. For 1 patient (with the greatest TV overlap with the PTV at 13.7%) and close proximity of the PTV to the spinal cord, better bone marrow sparing with VMAT62.5bm was impossible, without exceeding the dose constraints for the other organs at risk. The dose to the bones and TV was compared for each patient for SFO62.5 versus VMAT62.5 and SFO62.5 versus VMAT62.5bm (Fig. 4). The SFO62.5 plans compared with the original VMAT plans reduced the mean dose to the bones by a median of 4.5 Gy (IQR 3.6-5.4; Z = −4.015; *P*<.001) or a median of 3.1 Gy (IQR 2.6-3.6; *P*=.002) compared with the reoptimized VMAT62.5bm plans. The proton plans were better at sparing the low-dose region, even compared with the reoptimized VMAT plans, with a median 11.4% absolute reduction of bone V_10Gy_ (Z = −3.92; *P*<.001). For patients with a PTV >300 cm^3^, this reduction in the percentage of V_10Gy_ with protons was >7%.

The differences in the TV mean dose and TV V_20Gy_ as a function of the percentage of TV overlap for SFO62.5 versus VMAT62.5 and SFO62.5 versus VMAT62.5bm are also shown in Figure 4. The median gain in the TV mean dose with proton therapy versus the original VMAT62.5 plan was 4.6 Gy (IQR 2.9-6.4), which correlated with the percentage of TV overlap (Pearson correlation, *r*=−0.726; n=21; *P*<.001). However, on reoptimization (VMAT62.5bm plans), this difference in mean dose was a median of 0.6 Gy (IQR 0.1-1.5; *r*=−0.230; n=20; *P*=.329). For the TV V_20Gy_, SFO62.5 compared with VMAT62.5 gave an absolute difference of 11.6% (IQR 5.9%-18.9%), with correlated with the percentage of TV overlap (*r*=−0.796; n=21; *P*<.001). On reoptimization of the VMAT plans, the median reduction with SFO62.5 became 0.2% (IQR −1.9% to 1.3%) and did not appear to correlate with either the percentage of TV overlap (*r*=−0.125; n=20; *P*=.601) or PTV (not shown).

## Discussion

Our data have shown that in a representative group of patients with mid-esophageal cancer, bone marrow irradiation is highly dependent on the radiation treatment technique. We have demonstrated that using VMAT or protons can result in a reduction of the dose to bone and TV in the 20- to 30-Gy range compared with the 3D conformal treatment plans used in the SCOPE1 trial. Proton therapy plans offer the greatest potential for reduced irradiation of all thoracic bone structures, especially for patients with a larger PTV (>300 cm^3^ in the present study), and was especially useful in sparing the low-dose region at ∼10 Gy, which was not possible with the VMAT plans.

We have also demonstrated that significant TV sparing in the 20- to 30-Gy dose region is possible by reoptimizing the VMAT plans, even with dose escalation. Optimal treatment plans (using either protons or photons) would be best achieved using multicriteria optimization methods to balance dose sparing of the bone marrow with the dose constraints for the heart, lungs, and spinal cord for each individual patient, although this was outside the scope of the present study. The use of patient-specific beam arrangements for the proton therapy plans could also change the dose distribution in the region of the TV. Thus, the conclusions from the present study might not be applicable when using different beam geometries for treatment. Furthermore, proton plan robustness to respiratory and cardiac motion should be analyzed for different beam geometries, although this would require detailed information on both the timescale of the spot delivery and of each patient's breathing and cardiac motion. Our threshold limit of 300 cm^3^ for the PTV for the greatest potential benefit from proton therapy might also depend on the CTV and PTV margins used and might not be directly applicable to other target volume delineation protocols.

One of the limitations of the present study was the lack of consensus in the dose–volume parameters to predict HT, both in the organ-at-risk delineation and in the dose–volume threshold. We used values from the published data for thoracic tumors (non-small cell lung cancer and esophageal cancer), and although the values for the mean TV dose were similar (23.9-25.9 Gy), the values for TV V_20Gy_ varied much more (56.0%-70%).

Using the TV doses from Deek et al (22) as a threshold to predict leukopenia resulted in a variation of 19% to 38% for the 3D conformal plans, broadly in line with the 28% rate of HT observed in the standard arm of the SCOPE1 trial (2). The predicted toxicity rates for the VMAT (10%-29%) and SFO62.5 (0%-10%) plans were much lower. However, the cutoff values from Lee et al (23) predicted a much lower incidence of grade >3 leukopenia for all treatment techniques, with a maximum of 14% of 3D50 patients predicted to experience HT.

The study by Lee et al (23) and the present study had some notable differences. Also, the characteristics of the patient cohort included in the present study could have influenced the risk of HT, such as different chemotherapy regimens and different radiation therapy dose prescriptions and techniques. The clinical data from Lee et al (23) described preoperative esophageal cancer patients receiving 2 cycles of neoadjuvant chemoradiation therapy (cisplatin and 5-FU) to a maximum dose of 48 Gy. In contrast, the standard arm of the SCOPE1 trial prescribed 2 cycles of induction chemotherapy (cisplatin and 5-FU) before another 2 cycles of concurrent chemoradiation therapy to 50 Gy. The PTV in the study by Lee et al (23) was also much larger (median 519 cm^3^, range 300-1426) than in our study, possibly because of elective node irradiation, which might also affect the dose distribution in the region of the TV.

The use of different chemotherapy agents could have a significant effect on HT, and data from pelvic IMRT suggest that use of cisplatin and 5-FU causes significantly less HT than the use of mitomycin C (27). We would anticipate similar effects for thoracic tumors; thus, not only the chemotherapy agent, but also the timing and dosage of the chemotherapy regimen could alter the dose tolerance of the thoracic bone marrow.

The benefit of proton therapy and/or the reoptimization of photon plans depends on whether irradiation of all the bone in the thoracic region or just the body of the TV are most important for HT. Evidence from FLT-PET imaging has shown that the proportion and distribution of active bone marrow in the thorax varies considerably with age and gender (28). One limitation of our study was that we used the external bone contour as a surrogate for active bone marrow, although the distribution of the active marrow might be very different for each patient and would influence the active bone marrow-sparing possibilities for each individual patient. The idea of a compensatory response in nonirradiated bone has also been proposed (11), and sparing a sufficient bone marrow reserve from even low doses in the thoracic region would suggest that protons should be used, especially for patients with a small absolute bone volume or receiving intense concurrent chemotherapy regimens. Future work would ideally require imaging of active bone marrow for each patient, to identify the region of active bone marrow to be spared and to select those patients who would benefit sufficiently from reoptimized photon plans or those requiring proton planning to reduce their risk of acute HT.

## Conclusion

VMAT plans can reduce the thoracic bone marrow dose in the 20- to 30-Gy range for patients with mid-esophageal cancer but will irradiate a larger bone V_10Gy_ than will conformal plans. Reoptimized VMAT plans showed improved sparing of the TV volume; however, only proton plans showed significant sparing for the bone V_10Gy_ and bone mean dose, especially for patients with a larger PTV.

## Supplementary Data

Table E1
