# Supplementary material for: Potential of Proton Therapy to Reduce Acute Hematologic Toxicity in Concurrent Chemoradiation Therapy for Esophageal Cancer
Source: Int J Radiat Oncol Biol Phys. 2017 Nov 1;99(3):729–37. doi: 10.1016/j.ijrobp.2017.07.025 (PMC5612280; doi:10.1016/j.ijrobp.2017.07.025)
Supplement: Table E1 [file mmc1.pdf]

Dose-volume metric<br>Plan Comparison	Dose-volume metric<br>Median, (min-max)	Wilcoxon Signed rank test<br>Z; p value
Bone mean dose (Gy)		
3D50 vs VMAT50	**12.1 (6.0 – 16.7) vs 9.9 (5.5 – 14.3)**	*-3.98; <.001*
VMAT50 vs VMAT62.5	9.9 (5.5 – 14.3) vs 10.4 (5.5 – 14.7)	-3.48; .001
SFO62.5 vs VMAT62.5	**5.7 (2.6 – 9.3) vs 10.4 (5.5 – 14.7)**	*-4.02; <.001*
SFO62.5 vs VMAT62.5bm	**5.7 (2.6 – 9.3) vs 8.5 (5.0 – 12.8)**	*-3.92; <.001*
Bone V20Gy (%)		
3D50 vs VMAT50	**27.5 (7.5 – 37.3) vs 17.1 (8.0 – 29.0)**	*-3.98; <.001*
VMAT50 vs VMAT62.5	17.1 (8.0 – 29.0) vs 18.2 (7.8 – 29.4)	-1.56; .118
SFO62.5 vs VMAT62.5	**7.6 (2.9 – 14.4) vs 18.2 (7.8 – 29.4)**	*-4.02; <.001*
SFO62.5 vs VMAT62.5bm	**7.6 (2.9 – 14.4) vs 11.9 (4.2 – 21.6)**	*-3.58; <.001*
Bone V10Gy (%)		
3D50 vs VMAT50	33.4 (19.5 – 51.4) vs 35.3 (17.7 – 54.1)	-2.17; .030
VMAT50 vs VMAT62.5	35.3 (17.7 – 54.1) vs 37.9 (17.4 – 54.3)	-3.25; .001
SFO62.5 vs VMAT62.5	**23.0 (10.2 – 38.4) vs 37.9 (17.4 – 54.3)**	*-4.02; <.001*
SFO62.5 vs VMAT62.5bm	**23.0 (10.2 – 38.4) vs 34.0 (16.4 – 48.3)**	*-3.92; <.001*
TV mean dose (Gy)		
3D50 vs VMAT50	20.9 (11.8 – 33.1) vs 20.7 (12.6 – 28.7)	-2.85; .004
VMAT50 vs VMAT62.5	20.7 (12.6 – 28.7) vs 20.9 (12.6 – 27.4)	-3.18; .001
SFO62.5 vs VMAT62.5	**17.6 (6.2 – 24.0) vs 20.9 (12.6 – 27.4)**	*-4.02; <.001*
SFO62.5 vs VMAT62.5bm	17.6 (6.2 – 24.0) vs 17.5 (10.6 – 24.6)	-2.07; .038
TV V20Gy (%)		
3D50 vs VMAT50	**51.5 (31.4 – 79.8) vs 46.0 (35.0 – 70.3)**	-2.35; .019
VMAT50 vs VMAT62.5	46.0 (35.0 – 70.3) vs 47.5 (34.3 – 66.9)	-1.90; .058
SFO62.5 vs VMAT62.5	**36.7 (12.3 – 53.2) vs 47.5 (34.3 – 66.9)**	*-4.02; <.001*
SFO62.5 vs VMAT62.5bm	36.7 (12.3 – 53.2) vs 37.0 (16.4 – 51.6)	-.037; .970
TV V10Gy (%)		
3D50 vs VMAT50	54.3 (40.1– 81.0) vs 50.7 (41.2 – 79.2)	-2.277; .023
VMAT50 vs VMAT62.5	50.7 (41.2 – 79.2) vs 51.1 (41.6 – 79.0)	-2.520; .012
SFO62.5 vs VMAT62.5	47.1 (19.5 – 58.1) vs 51.1 (41.6 – 79.0)	*-3.945; <.001*
SFO62.5 vs VMAT62.5bm	47.1 (19.5 – 58.1) vs 45.5 (33.0 – 62.6)	-1.270; .204

Table 1: Dose-volume values for bone and TV volumes for the group of 21 mid-oesophageal cancer patients for 3D50 conformal, VMAT50, VMAT62.5 , VMAT62.5bm and SFO62.5 plans. Values in **BOLD** indicate differences of potential clinical significance (i.e. >2Gy in dose or >5% in V_{xGy}). Statistical test data are presented with no correction for multiple testing, but values in *italics* indicate p<.001
